# Visualization of Confined Electrons at Grain Boundaries in a Monolayer Charge‐Density‐Wave Metal

**DOI:** 10.1002/advs.202306171

**Published:** 2023-11-20

**Authors:** Yaoyao Chen, Yu Zhang, Wei Wang, Xuan Song, Liang‐Guang Jia, Can Zhang, Lili Zhou, Xu Han, Hui‐Xia Yang, Li‐Wei Liu, Chen Si, Hong‐Jun Gao, Ye‐Liang Wang

**Affiliations:** ^1^ School of Integrated Circuits and Electronics MIIT Key Laboratory for Low‐Dimensional Quantum Structure and Devices Beijing Institute of Technology Beijing 100081 P. R. China; ^2^ Advanced Research Institute of Multidisciplinary Sciences Beijing Institute of Technology Beijing 100081 P. R. China; ^3^ School of Materials Science and Engineering Beihang University Beijing 100191 P. R. China; ^4^ Institute of Physics Chinese Academy of Sciences Beijing 100190 P. R. China

**Keywords:** band bending, charge‐density‐wave metal, grain boundary, scanning tunneling microscopy

## Abstract

1D grain boundaries in transition metal dichalcogenides (TMDs) are ideal for investigating the collective electron behavior in confined systems. However, clear identification of atomic structures at the grain boundaries, as well as precise characterization of the electronic ground states, have largely been elusive. Here, direct evidence for the confined electronic states and the charge density modulations at mirror twin boundaries (MTBs) of monolayer NbSe_2_, a representative charge‐density‐wave (CDW) metal, is provided. The scanning tunneling microscopy (STM) measurements, accompanied by the first‐principles calculations, reveal that there are two types of MTBs in monolayer NbSe_2_, both of which exhibit band bending effect and 1D boundary states. Moreover, the intrinsic CDW signatures of monolayer NbSe_2_ are dramatically suppressed as approaching an isolated MTB but can be either enhanced or suppressed in the MTB‐constituted confined wedges. Such a phenomenon can be well explained by the MTB‐CDW interference interactions. The results reveal the underlying physics of the confined electrons at MTBs of CDW metals, paving the way for the grain boundary engineering of the functionality.

## Graphical abstract

Two types of 1D grain boundaries, that is, 4|4P and 4|4E mirror twin boundaries (MTBs), are synthesized in a charge‐density‐wave metal monolayer NbSe_2_. Both the confined electronic states and the charge density modulations at the MTBs are captured via scanning tunneling microscopy measurements. These results pave the way for the grain boundary engineering of the functionality.

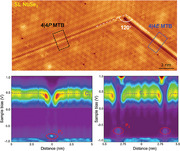

## 1 Introduction

Properties of atomically thin 2D materials are extremely sensitive to structural imperfections including defects, edges, wrinkles, and grain boundaries.^[^ ^1^, ^2^ ^]^ Precise characterization of atomic structures and electronic states at these structures has long been a pursuit of the nanoelectronics community. In the past decade, diverse structural imperfections have been widely studied in graphene, which are confirmed to realize rich physical phenomena, such as magnetic moments and topologically protected states.^[^ ^3^, ^4^, ^5^, ^6^ ^]^ Structural imperfections in transition metal dichalcogenides (TMDs) have been explored to a lesser extent, but are ubiquitous and supposed to generate more plentiful physics.^[^ ^7^, ^8^, ^9^, ^10^ ^]^ Among all, 1D grain boundaries in charge‐density‐wave (CDW) metals are of particular interest, because they provide ideal platforms for investigating the collective electron behavior in confined systems. However, clear identification of atomic structures at the grain boundaries, as well as precise characterization of the electronic ground states in CDW metals, have largely been elusive.

While 1D grain boundaries in TMDs are commonly reported in exfoliated flakes and chemical vapor deposition (CVD)‐grown samples, they mostly appear in isolation and remain defects or adsorbates,^[^ ^11^, ^12^ ^]^ which significantly limits the investigation of intrinsic electronic modulations around the boundaries. It is anticipating to controllably generate different types of atomic‐precise grain boundaries with specific interconnection. Very recently, molecular beam epitaxy (MBE) has been used for the growth of Mo‐based TMDs including MoS_2_ and MoSe_2_ with well‐defined 1D grain boundaries that are arranged into triangular networks.^[^ ^13^, ^14^, ^15^, ^16^, ^17^ ^]^ However, 1D grain boundaries have rarely been reported in non‐Mo systems, and to the best of our knowledge, have never been studied in CDW metals.

In this work, we provide direct experimental evidence of the confined electronic states at grain boundaries of MBE‐grown monolayer NbSe_2_, a representative CDW metal. Our scanning tunneling microscopy (STM) measurements, accompanied by the first‐principles calculations, reveal that there are two types of well‐defined grain boundaries in monolayer NbSe_2_, that is, 4|4P and 4|4E mirror twin boundaries (MTBs), both of which exhibit 1D boundary states and band bending effects. Moreover, the intrinsic CDW signatures of monolayer NbSe_2_ are efficiently suppressed as approaching an isolated MTB, but dramatically changed in the MTB‐constituted confined wedges. Such a phenomenon can be well explained by the MTB‐generated CDW interference. Our results present hallmark evidence of rich confined electronic states at MTBs, paving the way for grain boundary engineering of the functionality.

## 2 Results and Discussion

**Figure 1:**
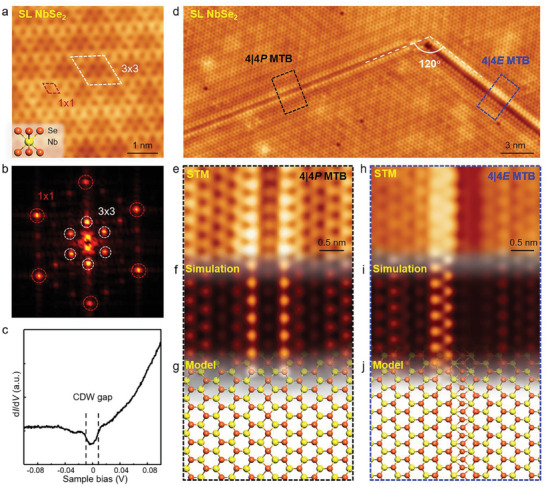
Atomic structures of MTBs in monolayer NbSe_2_. a) Atomically resolved STM image of pristine monolayer NbSe_2_ (*V*_s_ = −0.1 V, *I_t_* = 100 pA). The top Se atoms dominate the STM image, exhibiting a 3 × 3 CDW superlattice. Inset: Side view of the atomic structure of monolayer NbSe_2_. The yellow and orange spheres denote Nb and Se atoms, respectively. b) FFT image of panel a. The outer and inner six circles donate the atomic lattice and CDW superlattice of NbSe_2_, respectively. c) Typical STS spectrum of pristine monolayer NbSe_2_, exhibiting an obvious CDW gap pinning at the Fermi energy. d) Large‐scale STM image of monolayer NbSe_2_ (*V*_s_ = −0.3 V, *I_t_* = 100 pA). There are two types of MTBs, that is, 4|4P and 4|4E, with their intersection angle of 120°. e–j) Zoomed‐in STM images of 4|4P and 4|4E MTBs from panel d, as well as the corresponding STM simulations and atomic structures.

Monolayer NbSe_2_ is an iconic material of CDW states, hosting a nearly commensurate CDW superlattice when the temperature *T* <33 K.^[^ ^18^, ^19^ ^]^ **Figure** **1**a shows an atomically resolved STM image of MBE‐grown monolayer NbSe_2_ on graphene/SiC(0001) substrates acquired at *T* = 4.2 K (see Experimental Section). The topmost Se atoms of NbSe_2_ dominate the topography as bright protrusions and exhibit a 3 × 3 CDW superlattice aligned with the 1 × 1 atomic lattice, which is more evident from the corresponding Fourier transform image shown in Figure 1b. Moreover, the low‐energy scanning tunneling spectroscopy (STS) spectrum acquired on pristine monolayer NbSe_2_ exhibits a sharp dip pinning at the Fermi energy *E_F_* (Figure 1c), which is supposed to be the CDW gap.^[^ ^19^ ^]^

Structurally, there are usually two types of MTBs orienting along the zigzag direction embedded into TMDs, namely 4|4P and 4|4E MTBs, owing to the C_3_ symmetry of TMDs and the mirror symmetry of MTBs.^[^ ^20^, ^21^ ^]^ Specifically, 4|4P MTB contains fourfold rings sharing a point and is verified to be more common. In contrast, 4|4E MTB contains fourfold rings sharing an edge between them, which is rarely reported (Figure S1, Supporting Information). In our experiment, we successfully introduce a dense of both 4|4E and 4|4P MTBs in MBE‐grown monolayer NbSe_2_ islands with a ratio of ≈10:1. We find that the MTBs in monolayer NbSe_2_ islands are more likely to appear under a Se‐deficiency atmosphere, and the density of MTBs is positively associated with the Nb flux during the MBE process. For the maximal success yield, 21% of monolayer NbSe2 islands have 4|4E MTBs and only 2% of them have 4|4P MTBs. Such a result is well consistent with the ab initio thermodynamic simulations that the formation energy of grain boundaries in TMDs is extremely low.^[^ ^15^, ^22^ ^]^

In our as‐grown monolayer NbSe2 islands, 4|4E MTBs prefer to interconnect 4|4P MTBs with 120° relative orientation or appear in pairs with 60° relative orientation, as typically exhibited in Figures 1d and 3a, and Figure S2 (Supporting Information). Almost all the endpoints of MTBs extend to island edges. In such a case, the lengths of 4|4P and 4|4E MTBs are strongly dependent on the size of NbSe2 islands. In fact, the length of each 4|4E MTB that appears in pairs with 60° relative orientation varies from 7 to 30 nm. However, in contrast, the length of each 4|4E or 4|4P MTB that appears simultaneously with 120° relative orientation ranges from 2 to 35 nm. Moreover, both the 4|4P and 4|4E MTBs are quite stable by applying tip voltage pulses or heating up to 80 K during our measurements.

The atomic structures of MTBs in monolayer NbSe_2_ can be precisely confirmed by the combination of high‐resolution STM images and density functional theory (DFT) simulations. As visualized in Figure 1e–g, the 4|4P MTB displays a line of dark dots surrounded by two parallel lines of bright dots in an STM image (the dots indicate the locations of the topmost Se atoms), implying that the fourfold rings at the 4|4P MTB share a point at the Se sites. Moreover, the atomic structures on both sides of the 4|4P MTB are mirror‐symmetric, which are analogous to other TMDs.^[^ ^23^, ^24^ ^]^ In contrast, the 4|4E MTB exhibits a quite different STM topography. From Figure 1h–j, we can observe two parallel lines of bright dots next to each other at the 4|4E MTB, and the atomic structures on the two sides show a *π*‐phase shift. Such a characterization is in good agreement with the 4|4E MTB where the fourfold rings share a Nb edge between them. It's worth noting that, hitherto, few works have realized such a 4|4E MTB in monolayer TMDs.

**Figure 2:**
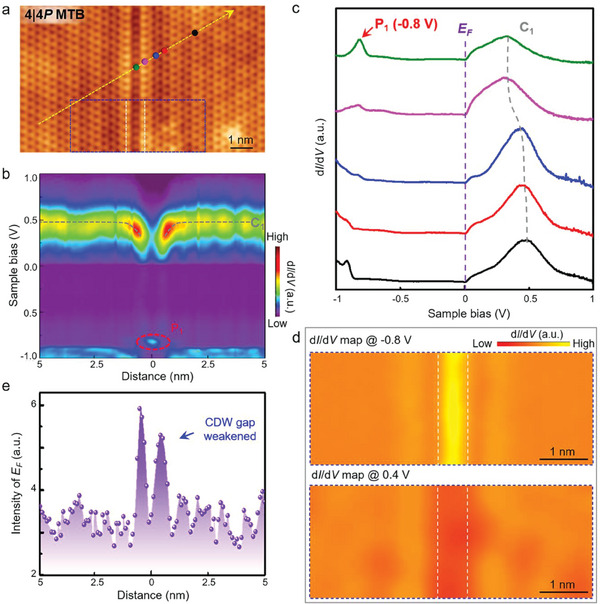
1D‐confined electronic states in 4|4P MTB of monolayer NbSe_2_. a) Atomically resolved STM image of an isolated 4|4P MTB in monolayer NbSe_2_ (*V*_s_ = −0.3 V, *I_t_* = 100 pA). b) Spatially resolved *dI/dV* spectra recorded along the yellow arrow marked in panel (a). The center of the 4|4P MTB is set as *x* = 0. When approaching the 4|4P MTB, the *C*_1_ peak of monolayer NbSe_2_ bends downward, together with the emergence of a P_1_ peak. c) Typical *dI/dV* spectra recorded on and off the 4|4P MTB, as marked by the corresponding color of the spheres in panel a. The *C*_1_ and *P*_1_ peaks, as well as the Fermi energy *E_F_*, are labeled accordingly. d) *dI/dV* maps recorded at the position marked by the blue rectangle in panel a at the sample bias of −0.8 and 0.4 V, respectively. The dotted lines donate the location of the two parallel topmost Se atoms. e) Spatially resolved *dI/dV* intensities at *E_F_* extracted from panel (b).

Now we concentrate on the electronic properties of monolayer NbSe_2_ modified by the MTBs. All the spectroscopic measurements are carried out under *T* = 4.2 K to reveal the local density of states (LDOS) and charge density distributions in the vicinity of MTBs. **Figure** **2**b shows the spatially resolved STS spectra recorded across an individual 4|4P MTB, as marked by the yellow arrows in Figure 2a, with several typical STS spectra extracted in Figure 2c and Figure S3(Supporting Information). From the figure, we can observe three notable signatures. First, the conductance band C_1_ of pristine monolayer NbSe_2_ gradually bends downward as approaching the 4|4P MTB within 1.0 nm, with the maximal bending energy *ΔE* of ≈0.17 eV. Such a band‐bending effect is in contradiction with MTBs or edges in 2D semiconductors such as MoS_2_, MoSe_2_, and WSe_2_, where the bands usually bend upward.^[^ ^25^, ^26^, ^27^ ^]^

Second, there is a pronounced LDOS peak P_1_ emerging at the energy *E* = −0.8 eV when recorded at the center of 4|4P MTBs in monolayer NbSe_2_. The spectroscopic maps further confirm the existence of charge accumulation within the energy range, as summarized in Figure 2d. By means of DFT calculations, the *P*_1_ is mainly verified to be attributed to the $d_{x^{2}-y^{2}}$ orbital of Nb atoms and *p_z_* orbital of Se atoms, which runs through the energy from −1.0 to −0.2 eV, as highlighted in Figure S4 (Supporting Information). Third and the most importantly, the STS spectra within the CDW gap are changed around the 4|4P MTB. By extracting the spatial evolution of the *dI/dV* signals at the *E_F_* shown in Figure 2e, we find there is an abrupt increase of *dI/dV* intensity as approaching the 4|4P MTB. Since the *dI*/*dV* intensity at the *EF* can roughly describe the CDW evolution (Figure S5, Supporting Information), our results demonstrate that the CDW signature of pristine monolayer NbSe_2_ are efficiently suppressed by an individual 4|4P MTB. In addition, the oscillation of the extracted *dI*/*dV* intensity at the *E_F_* is not periodic, and the relatively low intensity at the center of the MTB is likely to originate from the out‐of‐plane atomic reconstruction, as exhibited in Figure S6 (Supporting Information).

**Figure 3:**
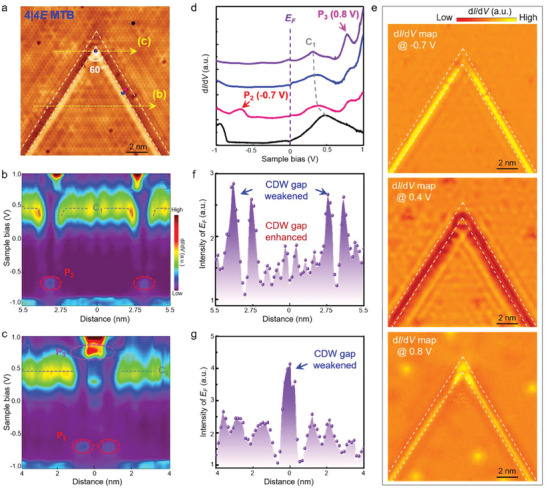
1D‐confined electronic states in 4|4E MTB of monolayer NbSe_2_. a) Atomically resolved STM image of two 4|4E MTBs in monolayer NbSe_2_ (*V*_s_ = −0.2 V, *I_t_* = 1 nA). The intersection angle between the two 4|4E MTBs is 60°. b,c) Spatially resolved *dI/dV* spectra recorded along the yellow arrow marked in panel a. The intersection of two 4|4E MTBs is set as *x* = 0. When approaching the 4|4E MTB, the *C*_1_ peak of monolayer NbSe_2_ bends downward, together with the emergence of *P*_2_ and *P*_3_ peaks. d) Typical *dI/dV* spectra recorded on and off the 4|4E MTB, as marked by the corresponding color of the spheres in panel a. The *C*_1_, *P*_2_, and *P*_3_ peaks, as well as the Fermi energy *E*_F_, are labeled accordingly. e) *dI/dV* maps recorded at the same position of panel a at the sample bias of −0.7, 0.4, and 0.8 V, respectively. f,g) Spatially resolved *dI/dV* intensities at *E_F_* extracted from panels b and c, respectively.

Similar electronic modulations, including the band bending effect, the emerging resonance peak, as well as the suppressed CDW signatures, also occur in the vicinity of an individual 4|4E MTBs in monolayer NbSe_2_. From the STS spectra shown in **Figure** **3**a–d, we can find out the conductance band *C*_1_ of pristine monolayer NbSe_2_ bends downward as approaching the 4|4E MTB (more STS spectra are given in Figures S7 and S8, Supporting Information). The spatial extension of the band bending effect is ≈1.2 nm and the maximal bending energy *ΔE* is ≈0.10 eV, slightly weaker than that of 4|4P MTBs. Moreover, there is an additional LDOS peak P_2_ appearing at the energy of −0.7 eV, which reaches the maximum at the center of 4|4E MTBs. To understand the origination of P_2_, we carry out the DFT calculations and confirm such a resonance peak is mainly attributed to the *p_z_* orbital of Se atoms, as given in Figure S9 (Supporting Information). It's worth noting that at the intersection point of two 4|4E MTBs (purple line in Figure 3d), an additional DOS peak P_3_ appears at the energy of 0.8 eV, which reaches the maximal intensity at the bright parallel lines exhibited in STM images. Our spectroscopic maps further confirm the electronic states of monolayer NbSe_2_ modulated by 4|4E MTBs, as summarized in Figure 3e and Figure S10 (Supporting Information). In addition, the CDW signature of pristine monolayer NbSe2 can be efficiently suppressed by 4|4E MTBs especially for the intersection point, as captured from the higher electron concentration at *E*_*F*_ in Figure 3c,d,g.

**Figure 4:**
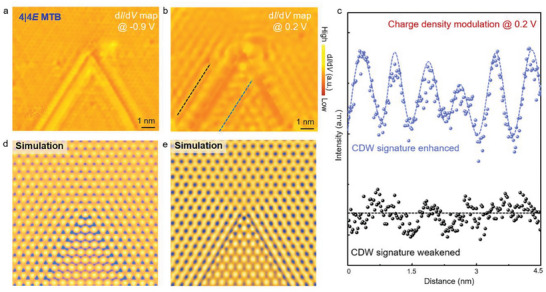
2D‐confined electronic states constructed by two 4|4E MTBs. a,b) Atomic‐resolution *dI/dV* maps recorded at the sample bias of −0.9 and 0.2 V, respectively. c) Line profiles acquired from the *dI/dV* maps along the dashed lines marked in panel b. The blue and black lines correspond to the charge density oscillations at the sample bias of 0.2 V inside and outside the MTB‐constructed regions, respectively. d,e) Toy models of 2D‐confined CDW states with different phases.

When two 4|4E MTBs interconnect each other with 60° relative orientation, there is a monolayer NbSe_2_ wedge formed by the two MTBs, as shown in Figure 3a. Such a construction provides an ideal platform to study the collective electron behavior, especially the CDW states, in confined systems. **Figure** **4**a,b shows typical spectroscopic maps of a monolayer NbSe_2_ wedge at the sample bias of −0.9 and 0.2 V, respectively. Apart from the boundary states at the MTBs, the charge density signatures of monolayer NbSe_2_ show obvious energy and spatial‐dependent behaviors. Outside and away from the MTBs, both the two spectroscopic maps exhibit clear 3 × 3 CDW superlattices, which can be regarded as pristine monolayer NbSe_2_. As approaching the MTBs, for the sample bias of −0.9 V in Figure 4a, there is almost no difference in 3 × 3 CDW signatures outside and inside the MTB‐constructed NbSe_2_ wedge. However, in contrast, for the sample bias of 0.2 V in Figure 4b, the 3 × 3 CDW signatures are strongly suppressed outside the wedge, and simultaneously, are enhanced inside the wedge. This phenomenon is more evident from the line profiles acquired from the spectroscopic maps of 0.2 V near and along the MTBs, as exhibited in Figure 4c.

To the best of our knowledge, such a CDW modulation effect in confined systems has never been observed before, which can be well understood by the MTB‐CDW interference interactions. Theoretically, the in‐plane 2D CDW states can be characterized by the sum of three individual periodic modulations of the charge density in real space, which are connected by a threefold rotation symmetry.^[^ ^28^, ^29^, ^30^ ^]^ For each periodic modulation Ψ  =  *Ae* ^*i*φ^, there is a specific phase φ depending on the reference point. In the absence of structural defects, φ of the periodic modulations in three directions are arbitrary, yielding a well‐ordered CDW state with threefold rotation symmetry. When taking MTBs into account, the momentum vector of the MTB‐induced scattered wavefunction changes from *q* to −*q*, yielding a nonzero phase shift with respect to the incident waves. In such a case, the charge density modulations are governed by the MTB‐induced multiple interference of the three periodic modulations incidenting from an STM tip pointing toward the MTBs and their reflection. Therefore, at specific energies, CDW states of monolayer NbSe_2_ on the interior of a confined wedge are expected to exhibit either enhanced or suppressed signatures, as simulated in Figure 4d,e by using different φ. More importantly, this result is feasible for other 2D CDW metals, revealing the physical mechanism of collective electron behaviors in confined systems.

## 3 Conclusion

In conclusion, we provide direct experimental evidence for the confined electronic states and the charge density modulations at MTBs of CDW metals. We successfully synthesize two types of MTBs in monolayer NbSe_2_, that is, 4|4P and 4|4E MTBs. Our measurements show that both MTBs can introduce local band bending effects and additional resonance peaks. Moreover, the intrinsic CDW signatures of monolayer NbSe_2_ are efficiently suppressed as approaching an isolated MTB, but can be either enhanced or suppressed in MTB‐constituted confined regions, depending on the electron energies. Such a phenomenon is well explained by the MTB‐CDW interference. Our results reveal the significance of MTB‐CDW interference in CDW metals, paving the way for the investigation of collective electron behavior in confined systems.

## 4 Experimental Section

### Sample Preparation

The bilayer graphene (BLG) was obtained by thermal decomposition of 4H‐SiC(0001) at 1200 °C for 40 min. NbSe_2_ layers were epitaxially grown on BLG/SiC(0001) substrates by evaporating Se and Nb from an electron beam evaporator and a Knudsen cell evaporator, respectively. The flux ratio of Se and Nb is more than 10:1 to guarantee a Se‐rich environment. The BLG/SiC(0001) substrate was maintained at 500 °C during the growth, followed by a post‐annealing process at 400 °C for 20 min.

### STM/STS Measurements

STM/STS measurements were performed using a custom‐designed STM system at 4.2 K under ultrahigh‐vacuum conditions (USM‐1300, Unisoku). An electrochemically etched tungsten tip was used as the STM probe, which was calibrated by using a standard graphene lattice and a Si(111)‐(7 × 7) lattice. The STS measurements were taken by a standard lock‐in technique with a bias modulation of 20 mV at 973 Hz.

### First‐Principles Calculations

Density functional theory (DFT) calculations were performed using the Perdew–Burke–Ernzerhof (PBE) functional in the generalized gradient approximation (GGA), as implemented in the Vienna Ab initio Simulation Package (VASP) software.^[^ ^31^, ^32^ ^]^ The electron‐ion potential was characterized using the projected augmented wave (PAW) method.^[^ ^33^ ^]^ The kinetic energy cutoff was set to 500 eV, and structural optimizations were performed until the forces reached values lower than 0.02 eV Å^−1^. To simulate the grain boundaries in the NbSe_2_ samples, 1D ribbon models were constructed. Their periodicities were applied along the direction of the grain boundaries, while the other two directions were set up with vacuum layers of >15 Å. In the ribbon structures, distances >20 Å were set between the MTB and the edge in order to eliminate their interactions. The 1 × 12 × 1 Monkhorst–Pack (MP) grids were used to segment the Brillouin zones of 1D ribbon models.^[^ ^34^ ^]^

## Conflict of Interest

The authors declare no conflict of interest.

## Author Contributions

Y.Y.C., Y.Z., and W.W. contributed equally to this work. Y.Z., H.J.G., and Y.L.W. coordinated the research project. Y.Y.C., Y.Z., X.S., L.G.J., C.Z., L.L.Z., X.H., H.X.Y., and L.W.L. synthesized the samples and performed the STM experiments. W.W. and C.S. performed the theoretical calculations. All authors were involved in discussions of this work.

## Supporting information

Supporting Information

## Data Availability

The data that support the findings of this study are available from the corresponding author upon reasonable request.
