# Supplementary material for: Visualization of Confined Electrons at Grain Boundaries in a Monolayer Charge‐Density‐Wave Metal
Source: Adv Sci (Weinh). 2023 Nov 20;11(37):2306171. doi: 10.1002/advs.202306171 (PMC11462295; doi:10.1002/advs.202306171)
Supplement: Supplementary file 1 — Supporting Information [file ADVS-11-2306171-s001.pdf]

ADVANCED SCIENCE
Open Access

# Supporting Information

for *Adv. Sci.*, DOI 10.1002/advs.202306171

Visualization of Confined Electrons at Grain Boundaries in a Monolayer Charge-Density-Wave Metal

*Yaoyao Chen, Yu Zhang*, Wei Wang, Xuan Song, Liang-Guang Jia, Can Zhang, Lili Zhou, Xu Han, Hui-Xia Yang, Li-Wei Liu, Chen Si*, Hong-Jun Gao and Ye-Liang Wang**

# Supporting Information

**Visualization of confined electrons at grain boundaries in a monolayer charge-density-wave metal**

*Yaoyao Chen, Yu Zhang*, Wei Wang, Xuan Song, Liang-Guang Jia, Can Zhang, Lili Zhou, Xu Han, Hui-Xia Yang, Li-Wei Liu, Chen Si*, Hong-Jun Gao, and Ye-Liang Wang**

**Image simulation of 4|4P and 4|4E MTBs**

The Nb-edge 4|4P MTB shows the Se-Nb four-fold rings sharing a point at Nb sites with substantially decreased Se-Se distances, as shown in Figures S1a and S1e. The Se-edge 4|4P MTB shows the Nb-Se four-fold rings sharing a point at Se sites, as shown in Figures S1b and S1f. The simulated STM image shows the expected difference in 4|4P MTBs between Se-edge and Nb-edge, while the Nb-edge MTB can be resolved as two atomic lines of bright Se without the atomic line of dark Se between them.

The Nb-edge 4|4E MTB shows the Se-Nb four-fold rings sharing edge, as shown in Figures S1c and S1g. The Se-edge 4|4E MTB shows the Nb-Se four-fold rings sharing edge, as shown in Figures S1d and S1h. The simulated STM images of 4|4E MTB provide the slight difference in the electronic contrast. From figure S1c we can see the atomic line of Se on the middle and left of the Nb-edge MTB can be resolved. However, the atomic lines of Se on Se-edge MTB can be resolved. The simulated STM image of the Nb-edge 4|4E structure is in good agreement with the experimental STM image by compared the contrast.

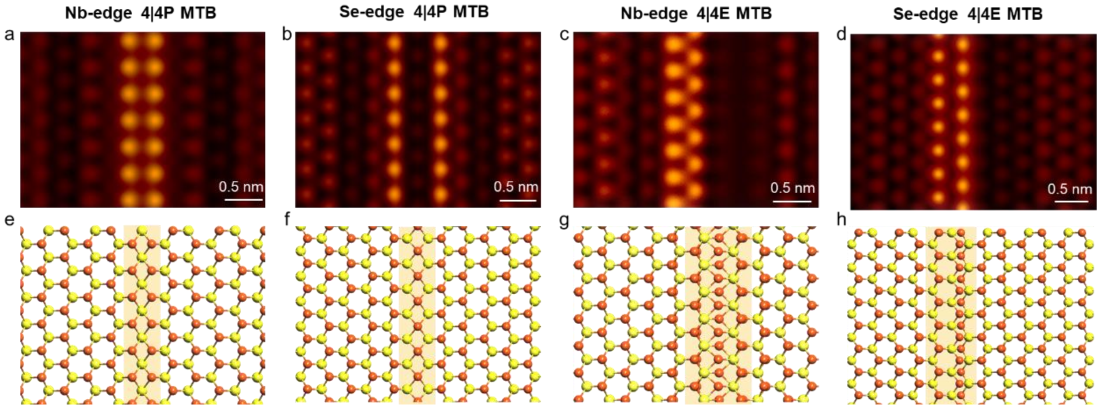


**Figure S1.** a,e) The simulated STM image and atomic structure of Nb-edge 4|4P MTB (shaded region). b,f) The simulated STM image and atomic structure of Se-edge 4|4P MTB (shaded region). c,g) The simulated STM image and atomic structure of Nb-edge 4|4E MTB (shaded region). d,h) The simulated STM image and atomic structure of Se-edge 4|4E MTB (shaded region). All the simulated STM images are acquired at the bias voltage of 1 V.

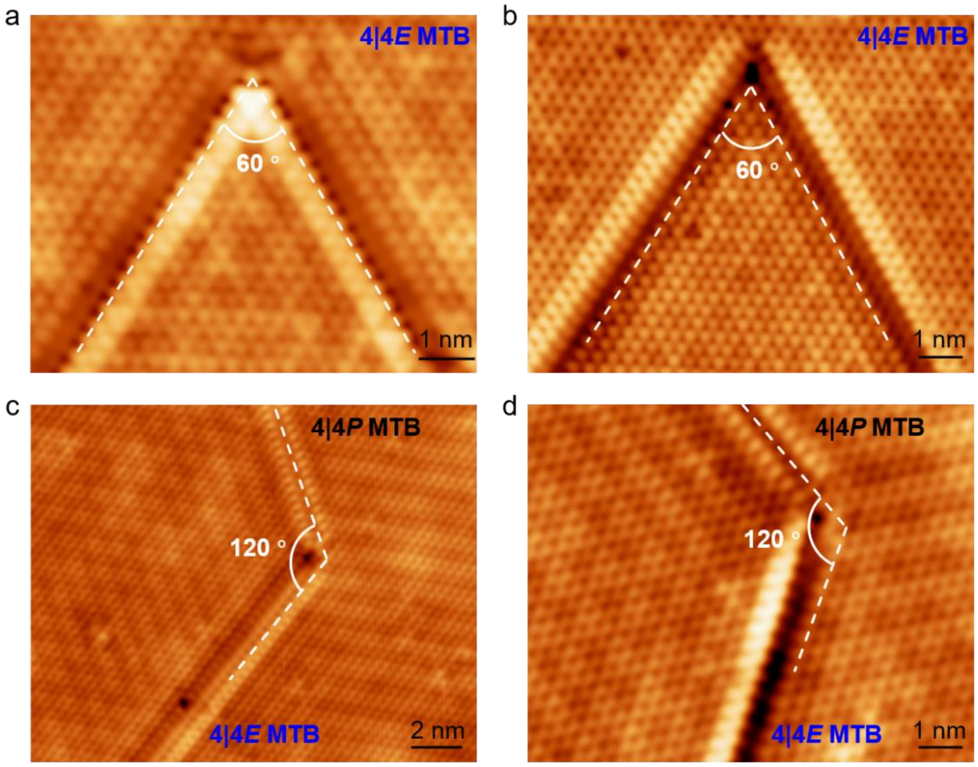


**Figure S2.** a,b) Typical STM images of two 4|4E MTBs with 60° relative orientation. c,d) Typical STM images of a 4|4E MTB and a 4|4P MTB with 120° relative orientation. Scanning parameters: (a) $V_s$ = -0.1 V, $I_t$ = 0.8 nA; (b) $V_s$ = -0.3 V, $I_t$ = 0.3 nA; (c) $V_s$ = -0.8 V, $I_t$ = 0.1 nA; (d) $V_s$ = -0.3 V, $I_t$ = 0.1 nA.

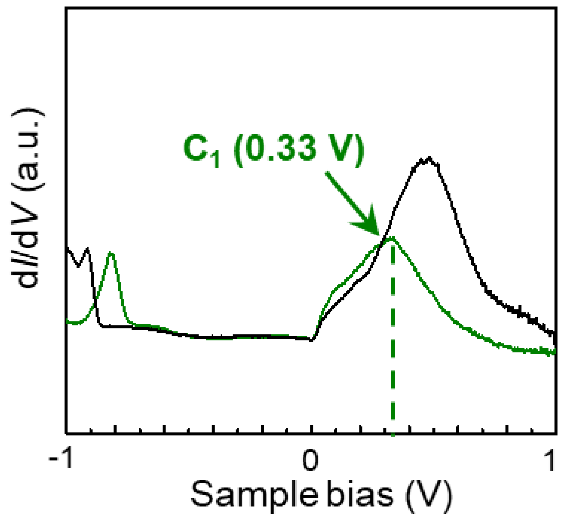


**Figure S3.** Typical STS spectra recorded at the pristine monolayer $NbSe_2$ (black line) and the center of the 4|4P MTB (green line), respectively.

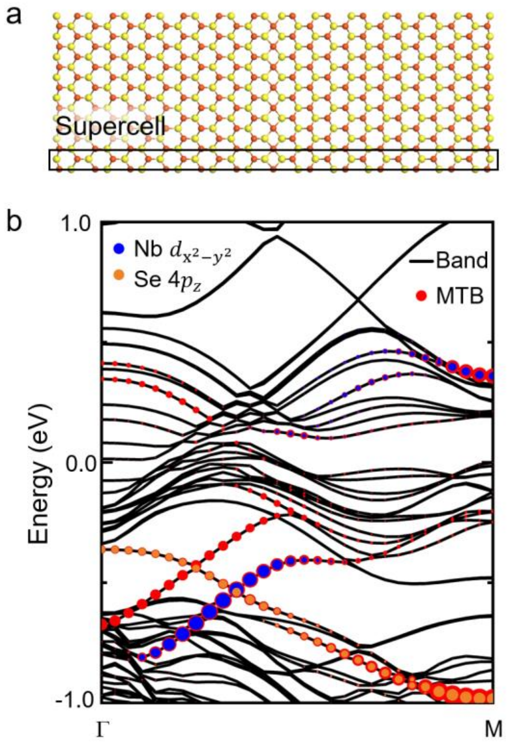


**Figure S4.** a) Atomic structure of the 4|4P MTB in monolayer $NbSe_2$. b) Calculated band structure of monolayer $NbSe_2$ with 4|4P MTB, where the states of the MTB are highlighted in red circles. The sizes of the blue and orange circles are proportional to the niobium $d_{x^2-y^2}$ and selenium $p_z$ characters, respectively.

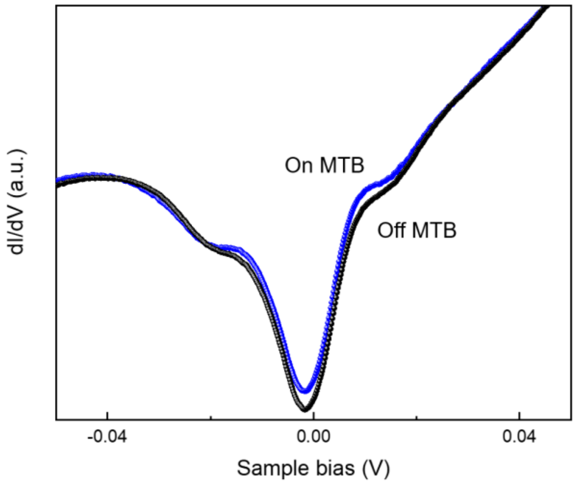


**Figure S5.** High-resolution STS spectra recorded on and off an MTB.

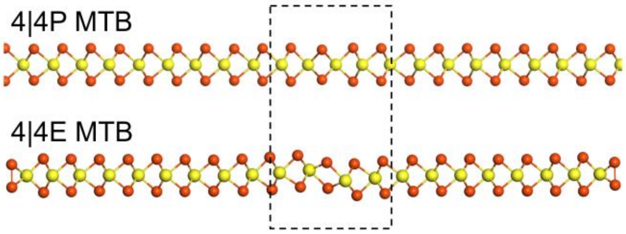


**Figure S6.** Side-view atomic structures of 4|4P and 4|4E MTBs in monolayer $NbSe_2$. With the consideration of atomic reconstruction, the topmost Se atoms at the center of the MTBs are significantly lower than that of pristine $NbSe_2$.

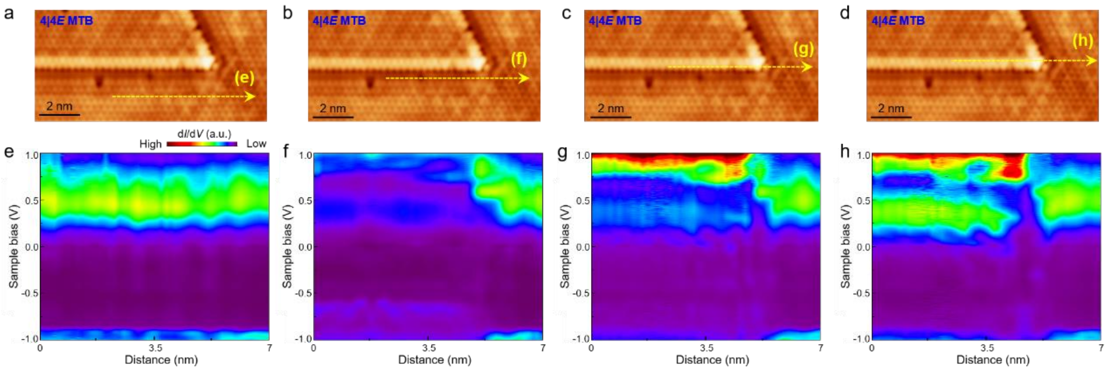


**Figure S7.** Typical STM image of two 4|4E MTBs in monolayer $NbSe_2$ interconnecting each other with 60° relative orientation. e-h) Spatially resolved *dI/dV* spectra recorded along the yellow arrows marked in panels a-d, respectively.

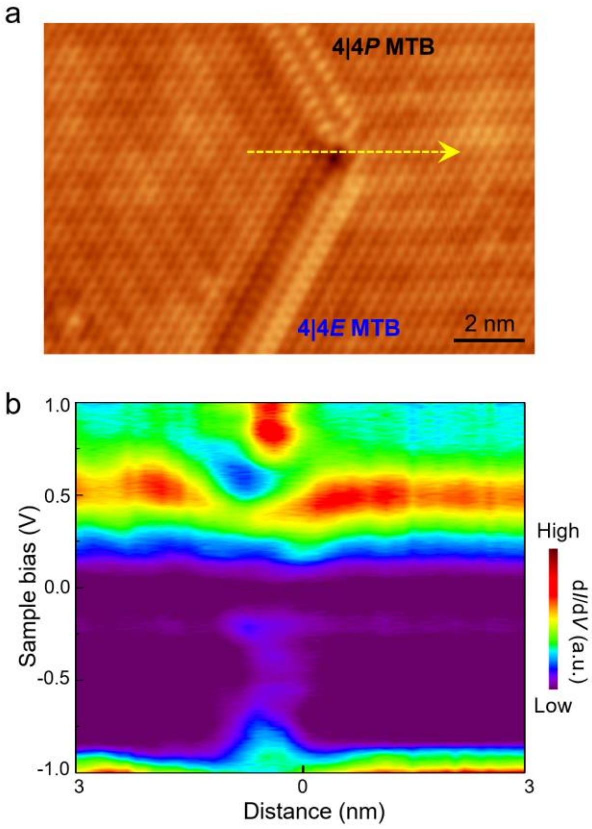


**Figure S8.** a) Typical STM image of 4|4E and 4|4P MTBs in monolayer $NbSe_2$ interconnecting each other with 120° relative orientation. b) Spatially resolved *dI/dV* spectra recorded along the yellow arrow marked in panel a.

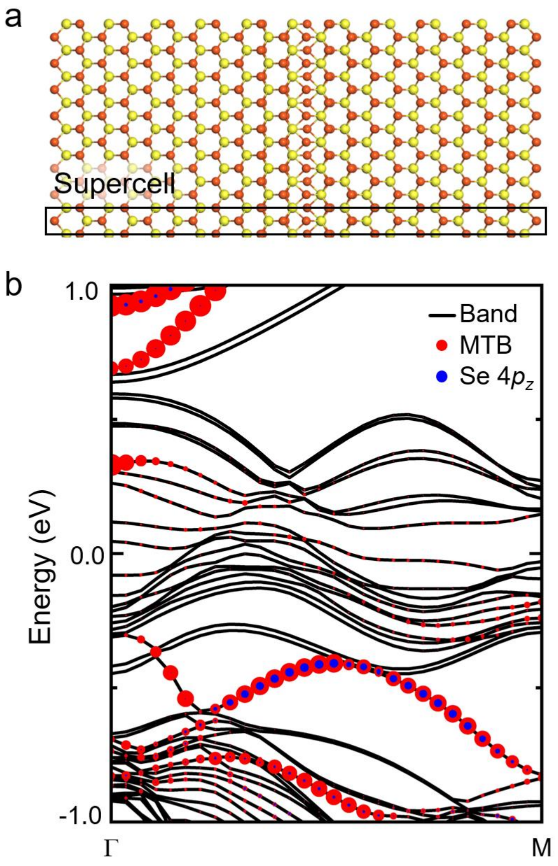


**Figure S9.** a) Atomic structure of the 4|4E MTB in monolayer $NbSe_2$. b) Calculated band structure of monolayer $NbSe_2$ with 4|4E MTB, where the states of the MTB are highlighted in red circles. The size of the blue circles is proportional to the selenium $p_z$ character.

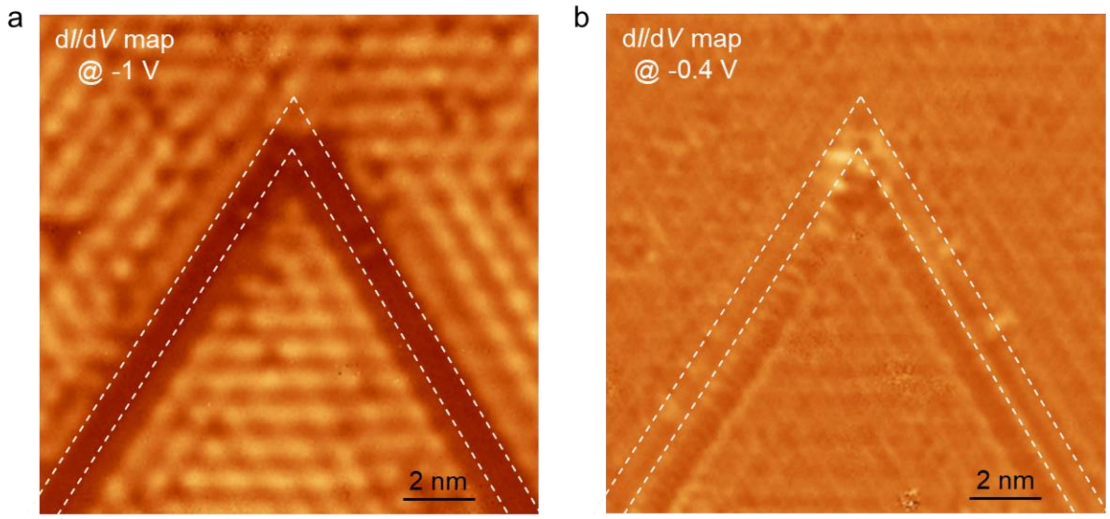


**Figure S10.** a,b) *dI/dV* maps recorded at the bias of -1.0 and -0.4 V, respectively.
